# Formulation and Characterisation of a Combination Captopril and Hydrochlorothiazide Microparticulate Dosage Form

**DOI:** 10.3390/pharmaceutics12080712

**Published:** 2020-07-30

**Authors:** Mellisa T. R. Chikukwa, Roderick B. Walker, Sandile M. M. Khamanga

**Affiliations:** Division of Pharmaceutics, Faculty of Pharmacy, Rhodes University, Makhanda (Grahamstown) 6140, South Africa; mtrchikukwa@gmail.com (M.T.R.C.); R.B.Walker@ru.ac.za (R.B.W.)

**Keywords:** captopril, hydrochlorothiazide, microparticles, sustained release, emulsion solvent evaporation, paediatric

## Abstract

Cardiovascular diseases such as hypertension and cardiac failure in South African children and adolescents are effectively managed long term, using a combination treatment of captopril and hydrochlorothiazide. The majority of commercially available pharmaceutical products are designed for adult patients and require extemporaneous manipulation, prior to administration to paediatric patients. There is a need to develop an age appropriate microparticulate dosing technology that is easy to swallow, dose and alter doses whilst overcoming the pharmacokinetic challenges of short half-life and biphasic pharmacokinetic disposition exhibited by hydrochlorothiazide and captopril. An emulsion solvent evaporation approach using different combinations of polymers was used to manufacture captopril and hydrochlorothiazide microparticles. Design of experiments was used to develop and analyse experimental data, and identifyoptimum formulation and process conditions for the preparation of the microparticles. Characterisation studies to establish encapsulation efficiency, in vitro release, shape, size and morphology of the microparticles were undertaken. The microparticles produced were in the micrometre size range, with an encapsulation efficiency >75% for both hydrochlorothiazide and captopril. The microparticulate technology is able to offer potential resolution to the half-life mediated dosing frequency of captopril as sustained release of the molecule was observed over a 12-h period. The release of hydrochlorothiazide of >80% suggests an improvement in solubility limited dissolution.

## 1. Introduction

The major contributors to non-adherence of treatment regimens include a limited understanding of medicines, forgetfulness, frequent dosing, pharmacological and organoleptic properties of the medicine such as adverse effects, dosage form size and palatability [1]. Efforts by health care providers and formulation scientists to address contributors to poor adherence may improve patient acceptability, adherence and ultimately, quality of life [1,2]. The majority of the medicines on the South African market are available as solid oral dosage forms, specifically tablets [3], which poses a challenge when treating paediatric and some geriatric patients with dysphagia. The approach to treatment in hospitals and pharmacies is to use extemporaneous compounding to prepare oral liquid dosage forms from tablets for administration to paediatric patients. Several concerns arise from the use of extemporaneous compounding including safety, efficacy and a lack of reliable clinical data [4,5]. Furthermore, conventional liquid dosage forms for oral use pose challenges with respect to stability, palatability and the cost associated with transport and storage of medicines. Consequently, there is a need to move towards the development of solid oral dosage forms (SODF) for use in this patient population. The benefits of SODF such as microparticulate or oro-dispersible dosage forms include dose flexibility and titration, ease of ingestion and enhanced patient adherence [6,7]. Efforts have been put into the development of multiparticulate dosage forms such as minitablets and microparticles [8,9]. Microparticles are solid dosage forms in which an active pharmaceutical ingredient (API) is incorporated into a polymeric matrix formed into spherical or irregular shaped particles between 1–1000 μm in size. Microparticles are classified as microspheres or microcapsules and in the latter case, the API is encapsulated with polymeric materials [10]. The use of microparticles and minitablets allows for localised, site specific release and absorption in the gastrointestinal tract (GIT), thereby improving efficacy and safety, and for uniform spread of particles throughout the GIT, resulting in constant plasma levels in comparison to when single unit dosage forms are used [11,12]. Minitablets (1–3 mm in diameter) are smaller than conventional tablets, making them easier to swallow for older children. Microparticles, however, may be used to increase the stability and bioavailability of an API compared to standard dosage forms and their small size makes them easier to swallow and spread throughout the GIT compared to minitablets [9,11,13,14]. Methods of microencapsulation such as phase separation and spray drying are more complex than emulsion solvent evaporation (ESE), which is a simple, easily scalable approach that results in lower residual solvent levels in particles [10,11]. The stability and activity of an API is not compromised due to the low temperatures and stirring speeds used when compared to other methods of microparticle manufacture [15].

The recommended regimen for the treatment of hypertension in the South African Paediatric Standard Treatment Guidelines (STG) and Essential Medicines List (EML), includes the use of an angiotensin converting enzyme (ACE) inhibitor, captopril (CPT), in combination with a diuretic, hydrochlorothiazide (HCTZ) [16,17]. The low aqueous solubility of 0.7 g/L of HCTZ [18] negatively impacts absorption resulting in variable bioavailability and increases the risk of toxicity to the gastric mucosa due to the presence of large amounts of unabsorbed API [19,20]. In order to improve the pharmacokinetic and pharmacodynamic profile of HCTZ, it is crucial to develop a drug delivery system (DDS) that is capable of enhancing solubility and subsequent dissolution to ensure better absorption and bioavailability [21,22]. CPT has a short half-life of approximately 1.6 h, requiring frequent dosing [3]. In addition, the unpleasant smell and taste attributed to the thiol functional group impacts adherence in paediatric patients. Furthermore, the instability of CPT in an aqueous environment makes it an ideal candidate for development as a SODF. These challenges can be overcome by designing a dosage form that exhibits sustained gastric delivery that exhibits taste masking capabilities to improve the palatability of CPT.

The aim of this study is to develop, formulate, manufacture and characterise a paediatric appropriate SODF with which the ability to sustain the release of CPT is achieved. In this way decreased dosing frequency and improved palatability are achieved whilst the use of a gastric dispersible formulation for HCTZ may improve the solubility limited dissolution of the compound.

## 2. Materials and Methods

HCTZ was purchased from Zheijang Menovo Pharmaceutical^®^ (Zheijang, China). CPT was donated by Protea^®^ Chemicals (Midrand, South Africa). Microcrystalline cellulose PH101 (MCC) was donated by FMC (Philadelphia, PA, USA). Eudragit^®^ E100 (Eud^®^ E100) and Eudragit^®^ RSPO (Eud^®^ RSPO) were donated by Evonik Rohm Pharma (GmbH, Darmstadt, Germany). Hydroxypropyl methylcellulose (HPMC) K15M and K100M were donated by Colorcon^®^ Limited (Kent, UK). Sodium starch glycolate (SSG) was donated by Aspen Pharmacare (Port Elizabeth, South Africa), and ethyl cellulose (EC) was donated by Aqualon (Delaware, DE, USA). Sodium alginate was purchased from Warren Chemical Specialties (Johannesburg, South Africa). Span 80 was purchased from Sigma Aldrich (Darmstadt, Germany) and liquid paraffin from Rochelle Chemicals (Johannesburg, South Africa). All other reagents were of analytical grade and were used without further modification.

### 2.1. Manufacture of Microparticles

#### 2.1.1. Manufacture of HCTZ Microparticles

**Figure 1:**
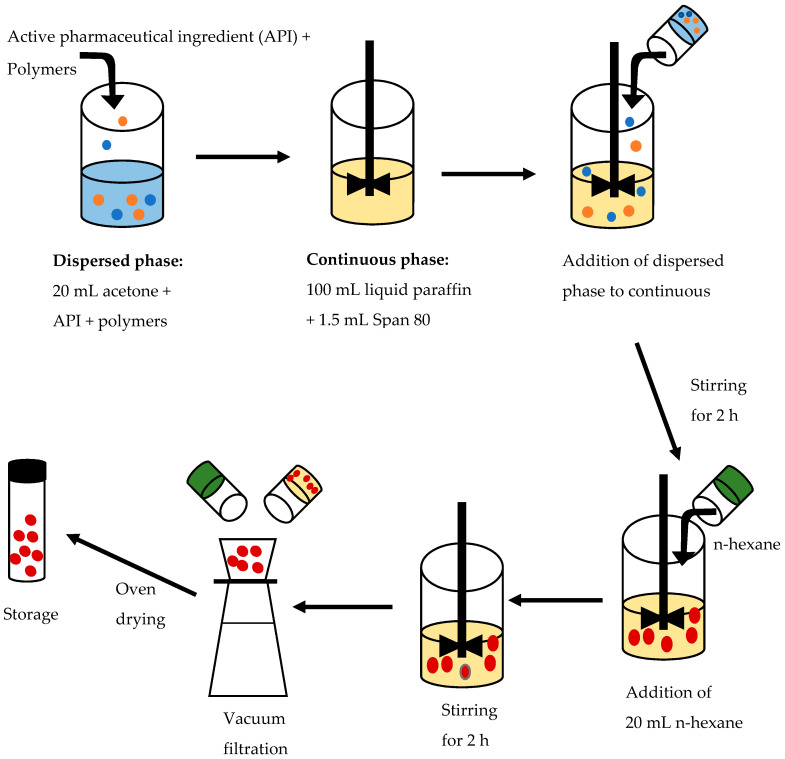
Schematic representation of the solvent evaporation process for the manufacture of hydrochlorothiazide (HCTZ) microparticles.

HCTZ and CPT microparticles were manufactured using ESE. A schematic representation of the manufacturing process is depicted in Figure 1. HCTZ microparticles were manufactured by dispersing 0.5 g HCTZ, 1.0 g MCC, 0.5 g HPMC K100M and different amounts of SSG and Eud^®^ E100 in a beaker containing 20 mL acetone. Light liquid paraffin (100 mL) containing 1.5% *w/v* Span 80 was added to a Teflon beaker (Lasec, Cape Town, South Africa) and agitated for 10 min using a digital overhead Model EURO-ST 40 D S000 stirrer (IKA Eurostar 40, Staufen, Germany) fitted with a four-blade propeller, to produce a homogenous oily phase. The HCTZ–polymer dispersion was added to liquid paraffin and stirred for 2 h at a pre-defined speed to facilitate the evaporation of acetone. A 20 mL aliquot of n-hexane was added to the system to harden the microparticles, after which stirring continued for a further 2 h. HCTZ, MCC PH101, HPMC K100M, liquid paraffin, acetone and n-hexane were maintained at a constant level for all experimental formulations. The microparticles were collected by vacuum filtration and washed three times with n-hexane to remove any residual liquid paraffin and were then dried overnight at 30 °C in an oven (Gallenkamp Limited, Leicestershire, UK) and finally stored in 50 g amber bottles at room temperature (22 °C).

#### 2.1.2. Manufacture of CPT Microparticles

The CPT microparticles were manufactured using a process similar to that reported in Section 2.1.1. Approximately 0.25 g CPT, 1.0 g MCC PH101, 0.5 g HPMC K15M, 1 g Eud^®^ RSPO, 0.5 g sodium alginate and different amounts of EC were dispersed in 20 mL acetone in a beaker. The polymer–CPT dispersion was added to a Teflon beaker containing 100 mL liquid paraffin and 1.5% *w/v* Span 80 and stirred for 3 h at pre-defined speeds to facilitate the evaporation of acetone. The amounts of CPT, MCC PH101, HPMC K15M, Eud^®^ RSPO, sodium alginate, liquid paraffin and acetone were constant for all formulations. The microparticles were collected using vacuum filtration and washed three times with n-hexane to remove any residual liquid paraffin after which they were dried overnight at 30 °C in an oven (Gallenkamp Limited, Leicestershire, UK). The particles were stored in 50 g amber bottles at room temperature (22 °C).

Design of experiments (DoE), specifically a Box–Behnken design (BBD) central composite design (CCD), was used to generate the experiments for the manufacture of the HCTZ and CPT microparticles, respectively, using Design Expert^®^ Version 12 statistical software (Stat-Ease, Minneapolis, MN, USA). The impact of formulation and process parameters on some critical quality attributes (CQAs) of the microparticles were investigated using these experimental designs. The input variables to be evaluated were identified in preliminary studies and are summarised in Table 1 with the outputs monitored *viz.* encapsulation efficiency (EE) and % API released. In combination with the observations made during manufacture, a numerical approach using Design Expert^®^ Version 12 statistical software (Stat-Ease, Minneapolis, MN, USA) was used to optimise the dosage form. Optimisation was conducted to identify the experimental conditions that met the target constraints set for the responses summarised in Table 1, namely, maximum encapsulation efficiency for CPT and HCTZ, maximum HCTZ released at 30 and 120 min and minimum CPT released at 30 and 480 min.

### 2.2. Characterisation

#### 2.2.1. Encapsulation Efficiency

Aliquots of microparticles equivalent to 25 mg HCTZ and 12.5 mg CPT were weighed into a 100 mL volumetric flask using a Mettler^®^ Toledo AG135 balance (Mettler Instruments, Zurich, Switzerland). The samples were made up to volume with 50 mL MeOH and 50 mL 0.1 M HCl and sonicated for 2 h using an ultrasonic bath (Ultrasonic Manufacturing Company, Krugersdorp, South Africa). A 10 mL aliquot of the solution was filtered using a 0.45 μm HVLP filter (Millipore Co., Bedford, MA, USA), then 1 mL of the filtrate was transferred to a 10 mL A Grade volumetric and made up to volume with HPLC grade water. The sample was analysed using a previously validated reversed phase high-performance liquid chromatographic (RP-HPLC) method [23]. The RP-HPLC system was comprised of a Waters^®^ Alliance Model 2695 separation module and a model 2996 PDA detector (Waters^®^, Milford, MA, USA) set at 210 nm and separation was achieved using a 5 μm BDS Hypersil^TM^ C_18_ 250 mm × 4.6 mm i.d. column (Thermo Fischer Scientific Inc., Johannesburg, South Africa) using a mobile phase of methanol: water in a 40:60 *v/v* ratio adjusted to pH 3.0 using 85% *v/v* orthophosphoric acid [23]. The encapsulation efficiency (% EE) was calculated using Equation (1).

(1) $$\%\;EE=\frac{\mathrm{actual}\;\mathrm{API}\;\mathrm{loaded}}{\mathrm{theoretical}\;\mathrm{API}\;\mathrm{loaded}\;}\times 100$$

#### 2.2.2. In Vitro Release

In vitro release studies were performed using a Hanson^®^ Vision G2 Elite 8^TM^ dissolution apparatus equipped with a Vision G2^TM^ Autosampler and AutofillT^M^ fraction collector (Hanson Research Corporation, Chatsworth, CA, USA). The dissolution test was undertaken using the apparatus in USP I mode with a dissolution medium of 900 mL, 0.1 M HCl (pH 1.2) and continuous stirring at 100 rpm. The dissolution bath and media were maintained at 37 ± 0.5 °C. Samples equivalent to 25 mg HCTZ and 12.5 mg CPT were placed into stainless steel dissolution baskets of mesh size #40. Aliquots (5 mL) were withdrawn at 15, 30, 60, 90 and 120-min intervals for HCTZ and 30, 60, 120, 240, 480 and 720-min intervals for CPT without medium replacement and filtered using a 0.45 μm HVLP filter prior to analysis using RP-HPLC.

#### 2.2.3. Powder X-ray Diffraction (PXRD)

PXRD analysis was conducted using a Bruker D8 Discover Diffractometer (Billerica, MA, USA) equipped with a proportional counter with Cu-Kα radiation of 1.5405 Å, a nickel filter at a voltage of 30 kV with an associated current of 40 mA. Samples were placed onto a silicon wafer slide and the data were collected in the 2*θ* = 10 to 50° range at a scanning rate of 1.5°/min with a filter time constant of 0.38 s and split width of 6 mm. Baseline correction was performed by subtracting a spline function fitted to the curved background. Version 14.0 evaluation curve fitting (EVA) software (XRD Commander Version 2.61, Bruker AXS GmbH, Karlsruhe, Germany) was used to reduce the data for analysis.

#### 2.2.4. Differential Scanning Calorimetry (DSC)

A Perkin Elmer 600 (Perkin Elmer^®^, Beaconsfield, UK) DSC fitted with a RC 90 refrigerated cooling system was used a heating rate of 20 °C/min and a nitrogen flow rate of 20 mL/min. Approximately 2.5–5 mg of each sample was weighed using a Mettler^®^ AG 135 top loading balance (Mettler Instruments, Zurich, Switzerland) into an aluminium pan that was sealed and placed onto the hot stage of the cell unit. The samples were heated using an empty aluminium pan as a reference. The data were analysed using version 11 Pyris™ Manager Software for Windows (PerkinElmer, Waltham, MA, USA).

#### 2.2.5. Fourier Transform Infrared Spectroscopy (FTIR)

Infrared absorption spectra of API, excipients and 1:1 *w*/*w* physical mixtures of API and excipient were generated using a Spectrum 100 FTIR ATR spectrometer (Perkin Elmer^®^, Beaconsfield, UK). A small amount of each sample was placed onto a diamond crystal and subjected to a force of approximately 100 N prior to analysis at a rate of 4 cm^−1^ (*n* = 6 scans) over the wavelength range of 650–4000 cm^−1^. The data were analysed using Peak^®^ version 4.00 spectroscopy software (Operant LLC, Burke, VA, USA).

#### 2.2.6. Scanning Electron Microscopy (SEM)

Particle shape, size and the surface morphology of API and excipients were evaluated using a Model TS Vega LMU Scanning Electron Microscope (Tescan, Brno, Czech Republic). Samples were placed onto double-sided carbon tape, adhered to carrier discs, 3 mm in height and 10 mm in diameter, and sputter coated with gold for 30 min under vacuum (0.25 Torr) using a sputter-coater (Balzers Union Ltd., Balzers, Lichtenstein). The samples were visualised at an accelerated voltage of 20 kV.

#### 2.2.7. Mathematical Modelling of In Vitro Release

Model dependent mathematical approaches were used to elucidate the mechanism of HCTZ and CPT release from the microparticles. The in vitro release data for the microparticles were fitted to zero-order, first-order, Higuchi and Korsemeyer–Peppas models using DDSolver, an add-in program for Microsoft Excel [24]. The model that best fitted the data was selected based on the coefficient of determination (*R*^2^) and the model with the highest R^2^ value was considered as the best fit model for this purpose.

## 3. Results and Discussion

### 3.1. Manufacture of Microparticles

Microparticles were manufactured using an oil-in-oil (*o*/*o*) ESE approach. Liquid paraffin and acetone were used at the continuous and dispersed phases, respectively with Span 80 to stabilise the emulsion, as reported in other experiments [25,26]. The use of dichloromethane DCM or methanol alone and mixtures thereof as the dispersed phase, failed to produce solid, appropriately sized microparticles. Acetone is a solvent with low risk to human health when used at normally acceptable levels of <50 mg/day as indicated in the International Conference of Harmonisation (ICH) Q3C (R6) guidelines [27]. Small, discrete microparticles were easily harvested for subsequent characterisation when produced from the experimental runs. Two batches failed to produce HCTZ microparticles in the micrometre size range.

#### 3.1.1. Manufacture of HCTZ Microparticles

During preliminary studies the impact of SSG, Ac-Di-Sol^®^ and Kollidon^®^ Cl on the release of HCTZ from microparticles was investigated in an attempt to enhance the dissolution rate by facilitating disintegration of the microparticles. Microparticles manufactured using Ac-Di-Sol^®^ and Kollidon^®^ Cl produced formulations that exhibited undesirable release profiles in low yield. SSG was therefore selected as the disintegrant for the manufacture of immediate release HCTZ microparticles. HCTZ microparticles were manufactured using Eud^®^ E100 as a matrix forming polymer.

The HCTZ microparticles manufactured in these studies were evaluated using response surface methodology (RSM). The effect of input variables on the responses monitored, was evaluated statistically using analysis of variance (ANOVA) and Design Expert^®^ Version 12 software (Stat-Ease, Minneapolis, MN, USA) and visualized using response surface plots. The responses observed in the experiments are summarised in Table 2.

Response surface plots and polynomial equations were used to visualise and mathematically represent the combined effects of the input variables on encapsulation efficiency and the % HCTZ released at 30 min and 120 min. ANOVA revealed that Eud^®^ E100 content and homogenisation speed had a significant effect on the EE and % HCTZ released at 30 min. Moreover, SSG content had a significant effect on the % HCTZ released at 30 min, while none of the input variables had a significant effect on % HCTZ released at 120 min. Increasing the amount of SSG resulted in increased viscosity of the solution thereby decreasing diffusion of HCTZ out of the polymer matrix resulting in a high EE. The absence of any hydrophobic end capping such as the presence of a free carboxylic acid in Eud^®^ E100 minimises hydrophobic HCTZ–polymer interactions resulting in a decrease in EE from 103.86 to 74.71% as the polymer concentration increased from 0.5 to 1.5 g [28]. Furthermore, a significant decrease in the EE was observed with a decrease in homogenisation speed. It is likely that the low turbulence observed at low agitation speeds may have resulted in loss of HCTZ from the dispersed phase resulting in a lower EE, as previously reported [29]. Increasing the Eud^®^ E100 content resulted in a slight decrease in the % HCTZ released at 30 min due to an increase in the thickness of the microparticle wall and consequently the diffusional path length of HCTZ as discussed in Section 3.2.4 [30,31,32]. Moreover, the solubility of Eud^®^ E100 in gastric fluids of pH <5 can be used to facilitate the release of an API from pharmaceutical formulations in the stomach [33,34]; therefore, an increase in the % HCTZ released at 30 min was observed with an increase in the Eud^®^ E100 content. SSG facilitates rapid disintegration of particles by absorbing the surrounding medium, swelling and disrupting the microparticles and dispersing into small fragments, thereby increasing the surface area of contact between HCTZ and the dissolution medium. This results in an increase in % HCTZ released at 30 min observed with an increase in SSG content [35,36,37]. The combined effects of homogenisation speed and Eud^®^ E100 content resulted in the greatest % HCTZ released when the two variables were set at a maximum level which may be due to the large surface area for release provided by the small particle size and rapid disintegrating effect of SSG.

ANOVA data revealed that the % HCTZ released at 120 min was not significantly affected by any of the input variables. This may be due to the large and significant amount of HCTZ released from the microparticles within 30 min of commencing the dissolution studies. Experimental runs, H-04 and H-06, produced large clumps of material outside the micrometre size range instead of discrete microparticles. Batch H-04 was made using the largest SSG:Eud^®^ E100 ratio at the lowest homogenisation speed resulting in agglomeration of the dispersed phase that prevented the formation of discrete microparticles [12]. Batch H-06 was made with the highest total polymer content at the lowest homogenisation speed which did not provide sufficient agitation necessary to facilitate complete evaporation of the organic solvent and maintain the dispersed phase as discrete globules, which would ultimately produce microparticles [12]. The output responses for the manufacture of HCTZ microparticles are summarised in Table 3 and include the best fit model and factors that had a significant impact on the outputs monitored.

#### 3.1.2. Manufacture of CPT Microparticles

In order to produce sustained release (SR) CPT microparticles, the inclusion of different rate controlling polymers in the formulation was investigated. The effect of different amounts of rate controlling polymer, such as Eud^®^ RSPO, Kollidon^®^ SR, Eud^®^ RS100, Eud^®^ RL100 and sodium alginate, on the responses monitored was investigated. Burst release of CPT was observed from microparticles manufactured with Kollidon^®^ SR, Eud^®^ RS100 and Eud^®^ RL100. Eud^®^ RSPO, however sustained the release of CPT from the microparticles sufficiently and was therefore included in further studies. A combination of hydrophilic and hydrophobic polymers was therefore used to tailor the release profile of CPT from these microparticles.

RSM was used to evaluate the performance of CPT microparticles and the effect of the input variables on the outputs monitored was statistically evaluated using ANOVA (Design Expert^®^ Version 12 software, Stat-Ease, Minneapolis, MN, USA). Response surface plots were used to illustrate the combined effects of EC content and agitation speed on the outputs EE, % CPT released at 30 min and % CPT released at 480 min. The % CPT release at 30 min was monitored to determine whether burst release of the water-soluble CPT from the microparticles, or surfaces thereof, had occurred. The sustained release performance of the formulation was determined by monitoring the % CPT released at 480 min. The experimental conditions and the outputs for the experiments conducted are summarised in Table 4.

EE was significantly affected by EC content, while CPT released at 30 min was significantly impacted by both EC content and homogenisation speed. With increasing amounts of polymer in the formulation available to encapsulate CPT, an increase in EE was observed. At low homogenisation speeds, the low turbulence and mixing energy were insufficient to ensure that CPT was retained as dispersed droplets, as previously reported by Sharma et al. [29]. Consequently, there was a decrease in EE as the speed of agitation decreased. A decrease in CPT released at 30 min was also observed as the homogenisation speed decreased and the EC content increased. The effect of homogenisation speed on the % CPT released at 30 min may have been due to the formation of microparticles with a low surface area thereby limiting CPT release. Furthermore, increasing the EC content increased the viscosity and thickness of the polymeric matrix and consequently the diffusional path length for CPT, resulting in a decrease in CPT release [38,39]. None of the input variables had a significant effect on the % CPT released at 480 min, however, increasing the EC content slightly decreased the rate of CPT release at this point. The decrease in the rate of % CPT released at 480 min was lower than the decrease observed at 30 min, possibly due to erosion of the polymer and subsequent decrease in the path length for diffusion of CPT over time [40].

The outputs monitored following the manufacture of HCTZ microparticles are summarised in Table 5 which also includes the best fit model and factors that have a significant impact on the responses monitored.

#### 3.1.3. Formulation Optimisation

The process and formulations were optimised to produce microparticles that met the initial predetermined specifications (Table 1). A numerical optimisation approach using Design Expert^®^ Version 12 software was used to identify the experimental conditions that would produce microparticles with the maximum EE, minimum % CPT release at 30 and 480 min, and maximum HCTZ released at 30 and 120 min. The batches with the highest desirability of 0.789 and 0.698, for CPT and HCTZ respectively, were manufactured and characterised. The accuracy of the optimisation process was evaluated by comparing the predicted and observed responses expressed as a % prediction error (PE) (Table 6).

The optimised HCTZ formulation (*n* = 3) produced a mean EE of 79.13% with 83.97% and 92.45% HCTZ released at 30 and 120 min, respectively. The 83.97% HCTZ released from the optimised microparticles was observed at 30 min, suggesting that the product can be classified as an immediate release product according to Food and Drug Administration (FDA) guidelines, which state ≥80% must be released at 30 min [41]. The % PE for particle size, and HCTZ released at 30 and 120 min were acceptable at <6%. The % PE for EE was 26.31% suggesting the poor predictive capability of the model for % EE.

The EE of CPT was at a maximum and the % CPT released at 30 min was at a minimum so as to reduce the loss of CPT whilst producing a formulation with little or no burst release of the compound. Furthermore, the % CPT released at 480 min was minimised in order to produce a formulation that would ensure that the release of CPT from the microparticles was sustained. The optimised formulation produced a mean EE of 76.272% with 24.97% CPT released at 30 min and 58.20% released at 480 min. All outputs monitored exhibited an acceptable % PE of <6% suggesting the good predictive capacity of the model for EE and % CPT released at 30 and 480 min. There is a need to further optimise the formulation composition in order to further sustain the release of CPT and minimise the high rate of release observed within the first 4 h of the dissolution profile, as shown in the dissolution profile.

**Figure 2:**
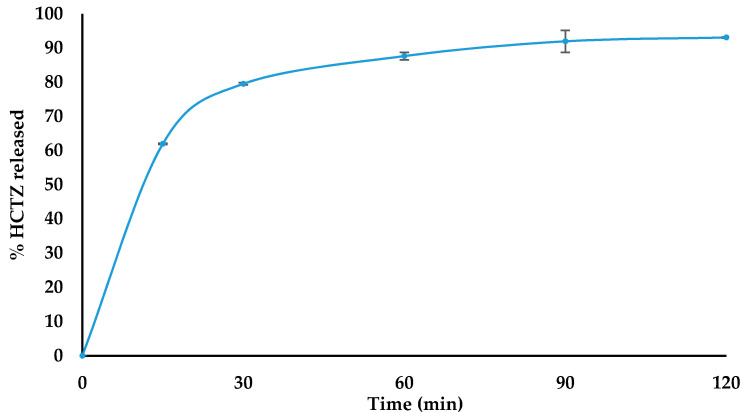
Dissolution profile (*n* = 6) of optimised HCTZ microparticles.

**Figure 3:**
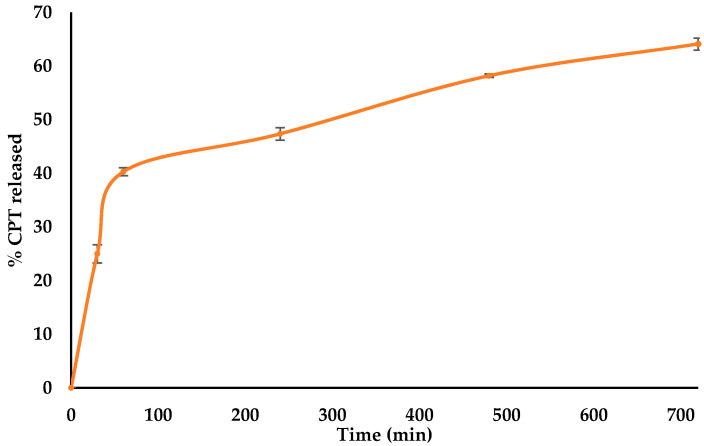
Dissolution profile (*n* = 6) of optimised CPT microparticles.

The dissolution profiles for the optimised HCTZ and CPT batches are depicted in Figure 2 and Figure 3, respectively. The daily recommended maximum paediatric doses for HCTZ and CPT are 0.2 mg/kg and 0.1 mg/kg, respectively [42]. Since each batch of CPT and HCTZ microparticles was manufactured with an amount of API equivalent to a single adult dose, it can be assumed that the amount of API released from the optimised batches is sufficient to result in a therapeutic effect for paediatric patients.

### 3.2. Characterisation

As part of ensuring the manufacture of a cost-effective and high-quality dosage form, preformulation studies were undertaken. Characterisation of the API facilitated an understanding of the thermal stability and crystallinity of the CPT. Any incompatibility between CPT and excipients used may result in instability and poor in vivo performance of a dosage form, hence these studies were conducted. The results generated from DSC and FTIR investigations revealed that no significant incompatibilities between HCTZ and CPT and the potential excipients existed, suggesting that formulation development and manufacturing studies could continue. However, there is a need to conduct real-time stability studies to evaluate long-term stability.

#### 3.2.1. Powder X-ray Diffraction (XRD)

**Figure 4:**
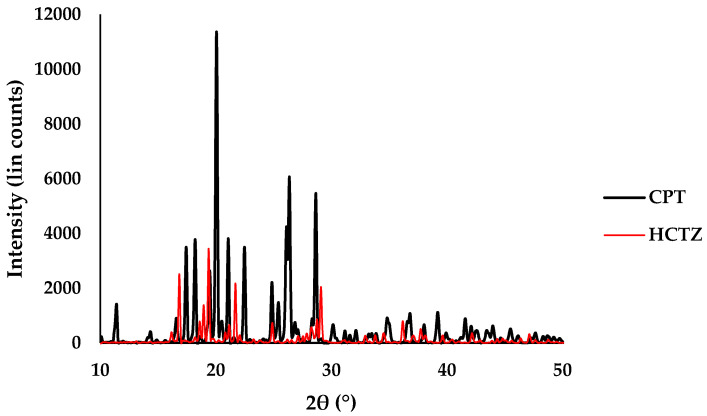
Powder X-ray diffractogram for HCTZ (red) and CPT (black).

XRD data studies were used to confirm the identify of HCTZ and CPT in correlation with the findings from DSC and FTIR studies, as well as to identify the crystalline nature of the API. The X-ray diffractogram depicted in Figure 4 confirmed the crystalline structure of HCTZ as is evidenced by the characteristic high-intensity peaks at 16.82°, 19.3°, 21.68 °, 24.94°, 29.12° and 36.14°. The X-ray diffractogram for CPT depicted in Figure 4 revealed the presence of high intensity peaks at 11.40°, 18.19°, 20.05°, 26.38° and 28.65° confirming the crystallinity of CPT. The experimental data were compared and found to be similar to previously reported data [43,44,45,46].

#### 3.2.2. FTIR

**Figure 5:**
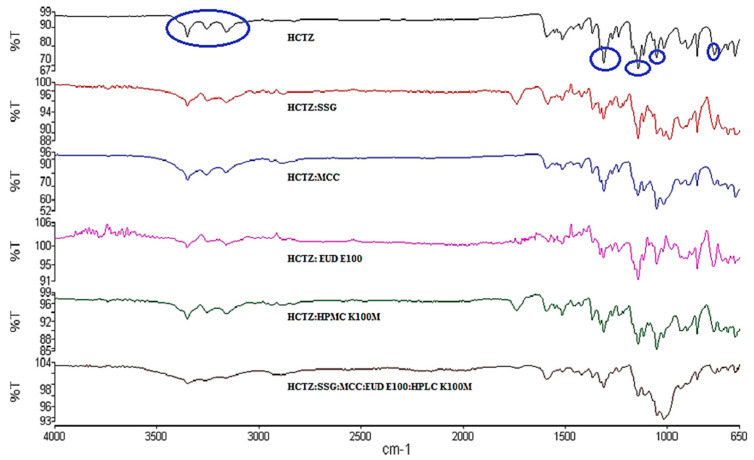
Fourier transform infrared spectroscopy (FTIR) absorption spectra of HCTZ and 1:1 mixtures of HCTZ and excipients.

The FTIR absorption spectrum of HCTZ (Figure 5) revealed prominent frequency vibrations at 3357 to 3165 cm^−1^ corresponding to NH stretching, 1315 cm^−1^ corresponding to asymmetric SO_2_ stretching, 1149 and 1057 cm^−1^ due to symmetric SO_2_ stretching and 771 cm^−1^ corresponding to C-Cl stretching [45,47]. The FTIR spectra of HCTZ and 1:1 *w*/*w* physical mixtures of HCTZ and potential excipients to be used for the manufacture of HCTZ microparticles are also depicted in Figure 5. The characteristic vibrational frequencies for HCTZ were visible in the spectra of the mixtures, with only slight shifts suggesting compatibility between HCTZ and excipients.

**Figure 6:**
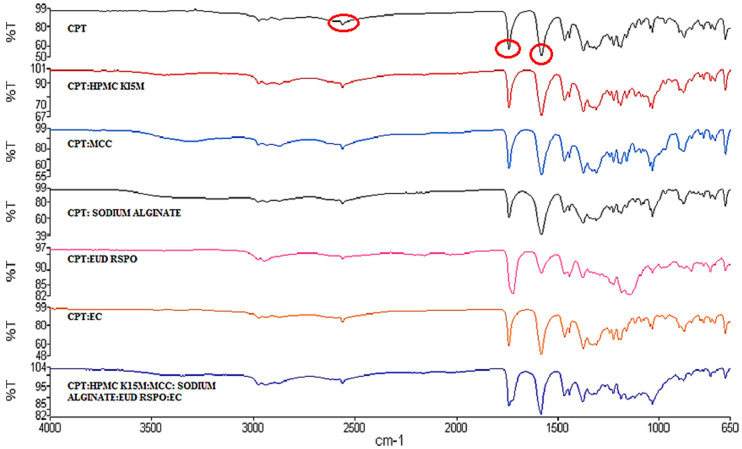
FTIR absorption spectra of CPT and 1:1 mixtures of CPT and potential excipients.

The FTIR absorption spectrum of CPT (Figure 6) revealed frequency vibrations at 2975 cm^−1^, 2562 cm^−1^, 1740 cm^−1^ and 1578 cm^−1^ representing C-H stretching, S-H stretching, C=O stretching of the carboxylic acid and C-N amide bond, respectively [48,49].

Moreover, the FTIR spectra of CPT and 1:1 *w*/*w* mixtures of CPT and potential excipients (Figure 6) to be used in the manufacture of the microparticles revealed no shifts in the characteristic vibrational frequencies for CPT suggesting compatibility between the API and excipients.

#### 3.2.3. DSC

**Figure 7:**
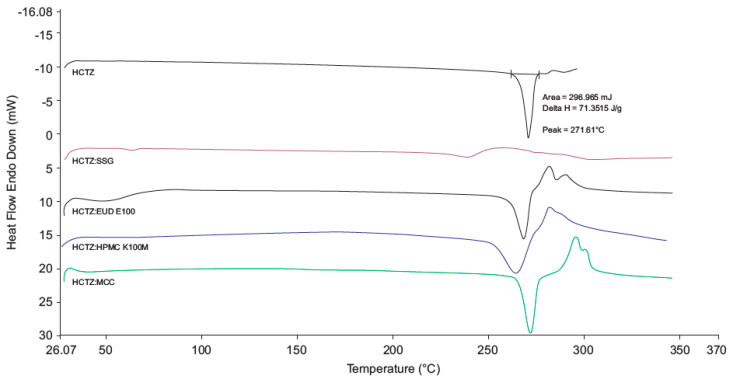
Differential scanning calorimetry (DSC) thermogram generated for HCTZ and 1:1 mixtures of HCTZ and potential excipients.

**Figure 8:**
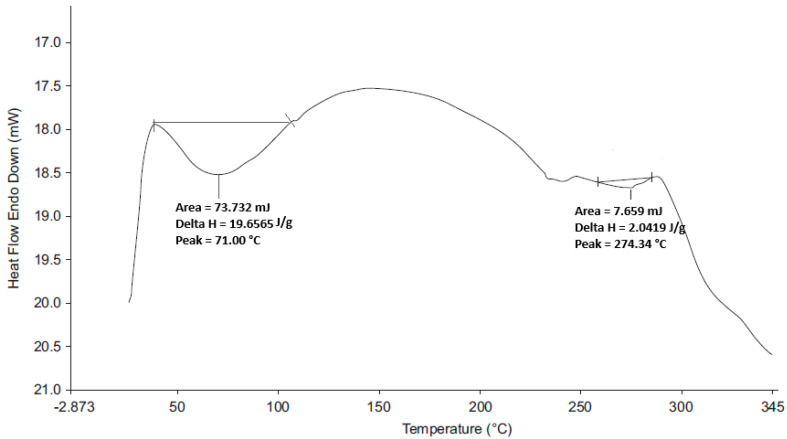
DSC thermogram of the optimised HCTZ microparticles.

The DSC thermograms for HCTZ and 1:1 *w*/*w* mixtures of the API and excipients (Figure 7) revealed a distinct endothermic peak at 271.61 °C, with a ΔH of 69.92 J/g, corresponding to the melting point of HCTZ [50,51]. The endotherm representing the melting point of HCTZ was visible in the DSC thermograms of HCTZ and excipient mixtures suggesting that HCTZ and the excipients tested are likely compatible. A slight shift in the endotherm was observed in the thermogram of the HCTZ:SSG mixture, which may have occurred due to decomposition of SSG which typically occurs at 250 °C [52]. The thermogram for the optimised HCTZ microparticles (Figure 8) revealed two broad melting endotherms. The first broad endotherm at 71 °C (ΔH = 19.66 J/g) is similar to that observed in the thermogram of a 1:1 mixture of HCTZ and Eud^®^ E100. The second endotherm at 274.34 °C (ΔH = 2.04 J/g) approximates the melting point of HCTZ. The absence of the sharp characteristic melting endotherm for HCTZ in the thermogram of the microparticles indicates that encapsulation of the API in the polymer matrix was achieved.

**Figure 9:**
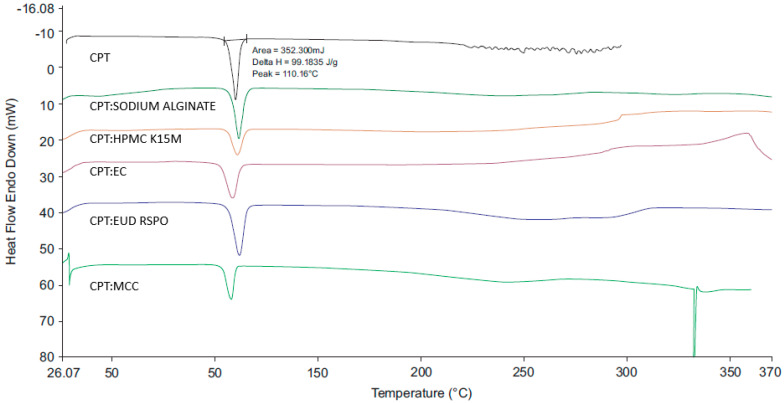
DSC thermogram for CPT and 1:1 *w*/*w* mixtures of CPT and potential excipients.

**Figure 10:**
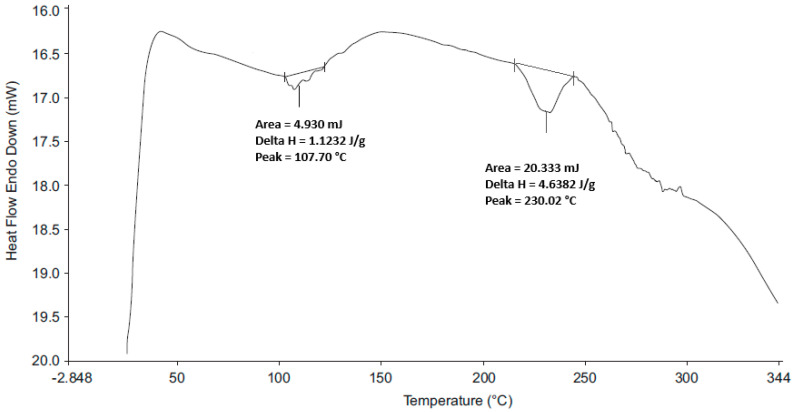
DSC thermogram of optimised CPT microparticles.

The DSC thermogram for CPT and 1:1 *w*/*w* mixtures of the API and excipients (Figure 9) revealed a distinct endothermic peak at 110.16 °C which corresponds to the melting point of the stable polymorph of CPT [44,48] with a ΔH of 99.18 J/g. The DSC thermogram observed for the optimised CPT microparticles (Figure 10) revealed a broad endotherm at 107.37 °C (ΔH = 0.42 J/g), close to the melting point of CPT and a second broad endotherm at 230.02 °C (ΔH = 4.63 J/g) that may be attributed to excipients present in the formulation.

#### 3.2.4. Scanning Electron Microscopy (SEM)

SEM micrographs of the HCTZ microparticles revealed that in most cases, close to spherical or oval-shaped particles were produced. The homogenisation speed and total polymer content was found to have a combined effect on particle size, whereas Eud^®^ E100 content affected the surface morphology of the product. At high homogenisation speeds, the high shear rates dispersed emulsion droplets effectively to form smaller droplets resulting in the production of smaller sized particles. The viscosity of the emulsion and droplets was high in formulations with a high polymer content resulting in the production of larger-sized droplets and ultimately microparticles. Batches produced at the lowest speed with a high Eud^®^ 100:SSG ratio failed to produce particles in the micrometre size range due to the absence of the necessary shearing rate to disrupt the viscous dispersed phase as it precipitated [53,54].

**Figure 11:**
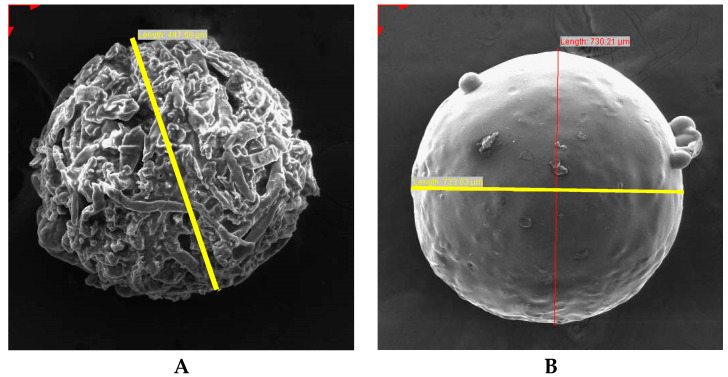
Scanning electron microscopy (SEM) micrographs of (**A**) optimised HCTZ (447.58 μm) and (**B**) CPT (730.21 μm) microparticles (yellow line indicates diameter).

SEM micrographs of the CPT microparticles revealed that all batches produced exhibited spherical particles ranging from 737.57 μm to 1261.99 μm in diameter. The surface morphology of the CPT microparticles was influenced by the amount of EC used, a hydrophobic polymer that is used for film coating or in polymeric matrices, in formulation. Batches manufactured with high EC content produced microparticles with smooth surfaces [55,56]. Conversely, batches produced with low EC content produced microparticles with rough, corrugated surfaces. In addition to EC content, batches manufactured at high homogenisation speeds produced microparticles with smooth surfaces due to more uniform mixing at high speeds as the high shear rate and uniform turbulence produced at high homogenisation speeds resulted in smaller-sized microparticles with smooth surfaces [53]. EC content and homogenisation speed were found to have an effect on the particle size. The particle size increased as the homogenisation speed decreased due to a decrease in the kinetic energy required to ensure the emulsion produced consisted of smaller droplets [53]. The increase in particle size was observed by increasing the EC content which may have been due to an increase in the viscosity of the emulsion with an increase in polymer content, resulting in large droplets in the dispersion and therefore large microparticles [38]. SEM micrographs of the optimized HCTZ and CPT microparticles are depicted in Figure 11.

#### 3.2.5. In Vitro Release Kinetics of HCTZ

The highest R^2^ values were obtained for the Korsmeyer–Peppas model suggesting HCTZ release was facilitated by erosion and dissolution of Eud^®^ E100 in media of pH < 5 [54]. The *R*^2^ values for the Higuchi model, which is diffusion-based, were close to those obtained for the Korsmeyer-Peppas model, indicating that it is likely that several mechanisms of release are involved [57]. HCTZ release from the microparticles is facilitated by disintegration/erosion of the polymers and diffusion of the poorly water soluble HCTZ from the water-soluble polymeric matrix. The formulation developed and reported herein, has the ability to improve the dissolution rate of HCTZ, however in vivo studies are required to evaluate if the rate of solution, permeability and consequently bioavailability improved.

The in vitro release data for CPT was best fitted by the Korsemeyer-Peppas model, however, high R^2^ values were also observed for the Higuchi and Hixson–Crowell models indicating that CPT release from the microparticles is likely to be a consequence of a several different mechanisms [57]. The release of CPT from the microparticles was found to occur via diffusion of CPT from swellable, non-eroding polymeric matrix produced with sodium alginate, HPMC and Eud^®^ RSPO, following release kinetic analysis. The technology has the ability to sustain the release of CPT over a 12-h period.

## 4. Conclusions

An age appropriate, paediatric microparticulate, solid oral delivery system with the capacity to reduce the dosing frequency and improve the taste of CPT was developed and characterised. In addition, the delivery system exhibited an increased dissolution rate of HCTZ suggesting an overall potential to increase the availability of HCTZ at the absorption site, when used in vivo. The size of the microparticles may reduce the difficulty in swallowing medicines reported in this target population. Furthermore, development of a microparticulate system allows for use of a weight-based dose titration without compromising the structural integrity and in vivo performance of the dosage form. There is potential for scale-up production, however gastric permeability of HCTZ and long-term stability of these microparticles should be investigated first. Furthermore, it would be beneficial to evaluate the palatability of the microparticulate system using electronic taste sensing systems as a CQA for the target population as done in our previous studies [58].

## Figures and Tables

**Table 1:** Input variables for the experimental design, outputs monitored and optimisation constraints.

API	Input Variables	Output Responses	Optimisation Constraints
**HCTZ**	SSG (g)	% EE	maximise
	Eud^®^ E100 (g)	% released at 30 min	maximise
	Speed (rpm)	% released at 120 min	maximise
**CPT**	Ethyl cellulose (g) Speed (rpm)	% EE	maximise
		% released at 30 min	minimise
		% released at 720 min	minimise

Abbreviations: HCTZ = hydrochlorothiazide; CPT = captopril; SSG = sodium starch glycolate; EE = encapsulation efficiency.

**Table 2:** Input variables and output responses monitored using a Box–Behnken design (BBD) for the manufacture of HCTZ microparticles.

Batch	Input Variables			Output Responses		
	SSG (A) g	EUD^®^ E100 (B) g	Speed (C) rpm	EE %	Released at 30 min %	Released at 120 min %
H-01	0.35	1.00	750	81.09	74.97	83.32
H-02	0.35	1.00	750	84.97	73.20	85.34
H-03	0.20	1.00	1000	91.00	75.11	85.03
H-04	0.20	1.00	500	70.30	60.00	60.81
H-05	0.20	0.50	750	85.15	75.70	90.53
H-06	0.35	1.50	500	70.14	62.38	63.52
H-07	0.35	0.50	1000	82.01	39.09	73.33
H-08	0.20	1.50	750	78.11	83.01	94.81
H-09	0.50	1.50	750	74.71	89.32	106.32
H-10	0.50	0.50	750	103.86	86.55	94.25
H-11	0.35	1.00	750	86.47	70.32	85.89
H-12	0.35	1.00	750	84.18	73.66	104.8
H-13	0.35	1.50	1000	83.76	84.24	89.62
H-14	0.35	1.00	750	89.81	72.42	88.80
H-15	0.50	1.00	500	81.42	73.65	98.29
H-16	0.50	1.00	1000	83.24	86.24	91.89
H-17	0.35	0.50	500	89.47	71.21	83.10

**Table 3:** Summary of outputs, the best fit models and significant input factors.

Output Response	Best Fit Model	Significant Factors
% EE	Quadratic	Eud^®^ E100
		Speed
% HCTZ released at 30 min	Quadratic	SSG
		Eud^®^ E100
		Speed
% HCTZ released at 120 min	2 FI	None

**Table 4:** Central composite design (CCD) input variables and outputs for the manufacture of CPT microparticles.

Batch	EUD^®^ RSPO g	Speedrpm	EE %	Released at 30 min %	Released at 480 min %
C-01	0.586	1000	30.75	58.27	86.20
C-02	3.00	1250	68.16	27.02	61.71
C-03	1.00	1250	43.28	53.99	66.60
C-04	2.00	1353.55	75.05	33.31	55.19
C-05	2.00	1000	64.60	33.96	69.83
C-06	2.00	1000	74.37	28.33	72.29
C-07	2.00	1000	68.34	30.21	77.52
C-08	2.00	646.45	55.25	7.38	40.03
C-09	2.00	1000	68.87	33.68	78.91
C-10	2.00	1000	70.26	26.22	74.63
C-11	1.00	750	70.51	33.82	73.41
C-12	3.414	1000	71.50	19.78	62.41
C-13	3.00	750	52.01	17.37	66.89

**Table 5:** Summary of responses and the best fit models and significant input factors.

Response	Best Fit Model	Significant Factors
% EE	Quadratic	Ethyl cellulose (EC)
% CPT released at 30 min	Quadratic	EC
		Speed
% CPT released at 480 min	Quadratic	None

**Table 6:** Comparison of predicted and observed outputs.

Batch	Output Monitored	Predicted Value	Experimental Value	Prediction Error (%)
H-OPT	% EE	100.00	79.13	26.31
	% released at 30 min	88.39	83.97	5.26
	% released at 120 min	88.37	92.45	4.41
C-OPT	% EE	79.28	76.27	−3.94
	% released at 30 min	24.82	24.978	0.65
	% released at 480 min	60.01	58.20	−3.11

Abbreviations: H-OPT = optimised HCTZ formulation, C-OPT = optimised CPT formulation.
